# The role of fever in febrile seizures: major implications for fever perception

**DOI:** 10.3389/fped.2023.1269205

**Published:** 2023-09-26

**Authors:** François Corrard, Robert Cohen

**Affiliations:** ^1^ ACTIV, Association Clinique et Thérapeutique Infantile du Val de Marne, Créteil, France; ^2^ AFPA, Association Française de Pédiatrie Ambulatoire, Talence, France; ^3^ Université Paris Est, IMRB-GRC GEMINI, Créteil, France; ^4^ GPIP, Groupe de Pathologie Infectieuse Pédiatrique, Paris, France; ^5^ Clinical Research Center (CRC), Centre Hospitalier Intercommunal de Créteil, Créteil, France

**Keywords:** fever, seizure, febrile seizure, afebrile seizure, children

## Introduction

Febrile seizures, the most common form of childhood seizures (1), are defined by the association of seizure with fever (2). For a long time and for many parents, fever has been a source of fear (3, 4), the main reason for concern being the possibility of seizure (3, 5). This is a real upset in the lives of parents: parents of nearly 9 out of 10 children who were present at the time of a first seizure said they experienced the death of their child (6, 7). The violent trauma is long-lasting, well beyond the first month, during which most parents experience a post-traumatic stress disorder (8). This syndrome, which is very intrusive, disrupts family ties, especially because it is sustained by the anxiety of reliving this experience until the child is 5 years old.

On average, one third of young children with a febrile seizure history experience seizure recurrence (9, 10). This presumed causality between fever and seizures, which is shared by nursing staff (11), leads to inappropriate and often exaggerated administration of antipyretics. This practice is all the more generalized because among the factors favoring the appearance of a crisis during a febrile time (young age, illness, genetic factors), only fever in reaction to illness can be controlled by medical treatment.”

The aim of this analysis was to try to determine the role of fever in triggering seizures in young children.

## Arguments for the responsibility of fever in triggering seizures

In addition to the concomitance of fever and seizures, in one study, the temperature of children measured before the seizure by parents or afterwards in the emergency department was found higher than that of a febrile control group without seizure (12). Furthermore, physiological arguments favor the causality of fever. Indeed, the increase in temperature increases neuronal excitation (13), and mutations of many genes involved in neuronal functioning are associated with febrile seizures. In particular, for the HCN2 mutation, the elevated temperature increases the rate of entry of positive ions into the axon, thus increasing its excitability (14).

## Arguments against the responsibility of fever in triggering seizure

First, infections associated with seizures are mainly viral (15). During a disease with digestive or respiratory symptoms, the same viruses can be associated with a febrile or afebrile seizure or both in the same child. In non-severe gastroenteritis, during which metabolic disorders cannot explain seizures (weight loss less than 5%, without electrolytes imbalance), seizures occur without fever, called “benign convulsion with mild gastroenteritis”, before or after the appearance of digestive signs (16). Four studies of children with rotavirus detected in the stool reported 80 children with febrile seizures and 142 with afebrile seizures (16–19). Furthermore, in the United Kingdom and Spain, the number of hospitalizations of children with seizures with or without fever decreased after rotavirus vaccination (20, 21). For another digestive virus, norovirus infections were associated with febrile seizures in 56 children and afebrile seizures in 64 (18, 22). In respiratory viral infection, the same virus was associated with febrile and afebrile seizures: H1N1 influenza virus (23–25), adenovirus (26), and respiratory syncytial virus (27, 28).

Second, febrile and afebrile seizures occurring during an infectious process are clinically similar (18). They both occur in healthy children, at the same age (from 6 months to 5 years), with the same peak between 13 and 24 months (9, 16), in both cases, 96% before 3 years of age (29). Both seizure types feature a sudden and total loss of consciousness, of the same short duration, most often less than 5 min (16, 17), and the same rather generalized presentation in 86% and 88% of febrile and afebrile types (29). The long-term prognosis for both types is similar and excellent without treatment (30–32). The 2 types of seizures may coexist in the same child. The few cohort studies reported that during the same episode of gastroenteritis, children presented a febrile seizure and then an afebrile seizure within 24 h (17). Conversely, other children who had an afebrile seizure presented a febrile crisis long afterward (31).

Third, some authors have already considered illness as more important than fever in triggering an attack. Certain afebrile seizures, called “provocated seizures”, occur without fever at the time of the seizure and are associated with definite symptoms or signs of minor infection. Lee et al. (33) noted 23% of these provoked seizures among 286 children with afebrile seizure. Zerr et al. (29) also distinguished “nonfebrile illness seizures” and found 36 cases of afebrile seizures or fever present during the week before the seizure. Patel et al. (34) and Wariuru et al. (35) associated febrile seizures with these afebrile seizures with delayed fever.

Fourth, the national German database of adverse events following vaccination was surveyed retrospectively for 3 consecutive years in children aged 0 to 6. Available clinical characterization identified 121 febrile seizures and 38 single afebrile seizures (36).

Fifth, the follow-up of children who had a febrile seizure, up to five years of age, shows that the average rate of recurrence is only about one third, even though febrile episodes are still frequent in this age group. In children with a genetic background compatible with the occurrence of a febrile seizure, fever, even high fever, is not sufficient to trigger a seizure.

Sixth, if the fever is high at the time of the seizure, the seizure only rarely occurs at the maximum fever peak (37).

Seventh, non-fever forms of epilepsy are associated with the same low-penetrance ion-channel gene mutations, including HCN2, as those seen in febrile seizures (38). Hence, in these same genetic conditions, fever is not necessary to trigger the seizures.

Eighth, a systematic review and meta-analysis of 8 studies found “weak evidence to suggest a possible role in preventing febrile seizure recurrence within the same fever episode and clearly no role for antipyretic prophylaxis in preventing febrile seizures during distant fever episodes” (39).

**Thus, fever is neither necessary nor sufficient to trigger a seizure in an infectious context**. This independence of the seizure from fever is the same as that of changes in the child's behavior that may occur during an illness (40). These 2 manifestations are collateral to the fever, more or less present and therefore more or less associated, without any direct link.

**If the trigger for seizure is not fever**, the trigger could be present in the infectious context. The inflammatory process induced by the child's immune response could play a role. As compared with simple febrile states, febrile seizures in this context are associated with a significant increase in levels of the inflammatory cytokines, interleukin 1 (IL-1), IL-4, IL-6, IL-10, HMGB1 and tumor necrosis factor (41–44). Under normal temperature conditions, injection of a high dose of IL-1 in a normal mouse triggered a seizure but had no effect in a mouse lacking IL-1 receptors (45).

## Conclusions

Fever is neither necessary nor sufficient to trigger a seizure during infectious diseases. This finding could help alleviate parental concerns and distress, particularly parents of children who have a seizure with fever. Parents and caregivers must understand that the aim of treatment is not to reduce fever or the risk of seizure but to reduce child discomfort in accordance with international recommendations.
